# DST: a Dual-path Swin Transformer framework for pig behavior recognition

**DOI:** 10.3389/fvets.2026.1868422

**Published:** 2026-07-14

**Authors:** Wangli Hao, Yujie Zhang, Hao Shu, Meng Han, Jiali Su, Qingqing Li, Fuzhong Li

**Affiliations:** 1 Faculty of Software Technologies, Shanxi Agricultural University, Jinzhong, Shanxi, China; 2 School of Information Science and Engineering, Shanxi Agricultural University, Jinzhong, Shanxi, China

**Keywords:** complex pigpen environments, decoupled spatial-channel attention, Dual-path Swin Transformer, frequency-domain fusion filter module, pig behavior recognition

## Abstract

Pig behavior recognition is a crucial component of intelligent animal health monitoring. In complex pigpen environments, traditional vision-based methods often exhibit poor feature robustness and deficient spatial-channel dependency modeling, two key limitations that compromise reliable performance. To mitigate these limitations, this study proposes a Dual-path Swin Transformer (DST) framework. This framework consists of two complementary paths for feature learning: a frequency-domain path and a spatial-domain path with enhanced channel modeling. In the frequency-domain path, a novel Frequency-domain Fusion Filter Module (FFM) is introduced. The Ideal Low-pass Filter extracts coarse-scale global structural features, while the Gaussian High-pass Filter captures fine-grained local edge features. In the spatial-domain path, an effective Decoupled Spatial-Channel Attention (DSCA) mechanism is developed. The spatial attention branch adaptively enhances features in key regions, and the channel attention branch automatically strengthens the weights of feature channels highly correlated with pig behaviors. The proposed DST is validated on a dataset containing 2,755 video clips covering six typical behaviors. Results show that DST achieves a recognition accuracy of 94.94%, which is 1.45 percentage points higher than the baseline Swin Transformer. These findings demonstrate that DST provides an effective and robust solution for automated pig behavior monitoring in complex agricultural environments.

## 1 Introduction

Pig behavior recognition is pivotal for achieving precision feeding, disease early warning, and welfare assessment in smart farming (1–4). In real-world piggery environments, however, high stocking density, frequent occlusions, individual phenotypic variability, variable lighting conditions, and cluttered backgrounds pose significant challenges to the accuracy and reliability of automated pig behavior recognition systems (5–7).

Traditional approaches, including manual observation and contact sensors, are impractical for large-scale deployment. Manual observation is subjective and labor-intensive (8–10), while wearable or implantable sensors risk interfering with natural behaviors and inducing stress responses (11–14). Non-contact vision-based methods have consequently become the mainstream direction for automated pig behavior monitoring.

Deep learning has driven substantial progress in this field. Convolutional neural networks (CNNs) have provided an important technical basis for vision-based livestock monitoring. For example, ConvNeXt enhanced the representational capacity of convolutional architectures through large-kernel convolutions and inverted bottlenecks (15). In livestock production scenarios, Pan et al. proposed a ResNet-based low-cost livestock sorting information management system with WGAN-assisted image enhancement, demonstrating the effectiveness of CNN-based visual recognition for pig recognition, growth-stage classification, and disease recognition under farm conditions, while also indicating that illumination variation and occlusion remain critical factors affecting recognition robustness (16). To capture temporal dynamics, researchers integrated CNNs with Recurrent Neural Networks (RNNs) or attention mechanisms for spatiotemporal feature fusion (17). Lightweight networks combined with attention modules were further proposed to handle occlusion and multi-view scenarios (18). Convolutional network designs have also been advanced via modernized densely connected architectures, where concatenation-based shortcuts exhibit superiority over conventional additive shortcuts in parameter efficiency and representational capacity (19). To address receptive field limitations, multi-level aggregation modules are adopted to expand the effective receptive field while maintaining efficiency (20). Furthermore, integration of biomimetic Overview-first-Look-Closely-next attention and Context-Mixing Dynamic Convolution empowers pure CNNs to model top-down attention and achieve state-of-the-art visual performance (21). Despite such progress, CNN-based methods still rely heavily on spatial features, restricting their ability to isolate task-relevant information from complex background noise.

The emergence of Vision Transformers (ViT) introduced global self-attention and improved the modeling of long-range dependencies (22). However, their high computational complexity and limited spatial inductive bias still restrict deployment in resource-constrained settings (23). The Swin Transformer alleviates these limitations through hierarchical feature maps and shifted window attention, achieving linear complexity while maintaining strong performance (24). To solve the low-rank problem of output features in linear attention and the redundancy of attention maps, Rank-Augmented Linear Attention and Partial Attention were proposed by Fan et al. and Vo et al., respectively (25, 26). Inspired by the sparse scanning mechanism of human vision, Zhang et al. designed a local modeling strategy based on Anchors of Interest (27). Together, these approaches break through the computational bottlenecks of Vision Transformers from different angles. They provide new ideas for building efficient vision backbones.

Although existing pig behavior recognition methods have achieved promising performance, poor feature robustness and insufficient modeling of spatial-channel dependencies remain in complex environments. To address these limitations, a Dual-path Swin Transformer (DST) framework is proposed in this paper. The frequency-domain path preserves global structural and local fine-grained features to enhance model robustness. Effective representations of key spatial regions and discriminative channels are captured by the spatial-domain path, which adaptively enhances the behavior-discriminative features of pigs and strengthens the model's representation capability. The main contributions of this paper are as follows:

- We propose a pig behavior recognition framework termed DST, which achieves collaborative feature extraction and modeling in both the frequency domain and the spatial domain. The frequency-domain path extracts global structural and local edge features via the FFM, enhancing the model's anti-interference capability in complex scenes. The spatial-domain path is based on the DSCA mechanism, which strengthens key regions and discriminative channel features, and accurately models cross-dimensional feature dependencies.
- We innovatively design an FFM, which adopts a complementary parallel architecture consisting of Ideal Low-pass and Gaussian High-pass filters. It extracts robust global structural features and fine-grained local detail features respectively, effectively improving the model's ability to suppress complex interferences such as illumination variations and object occlusions.
- This paper proposes a novel DSCA mechanism to accurately characterize the body posture and key behavioral features of pigs. Specifically, spatial attention adaptively focuses on key body regions of pigs. Channel attention adaptively enhances the weights of feature channels that are highly correlated with pig behavior. The collaboration of these two branches enables precise modeling of cross-dimensional feature dependencies.

## 2 Methods

**Figure 1:**
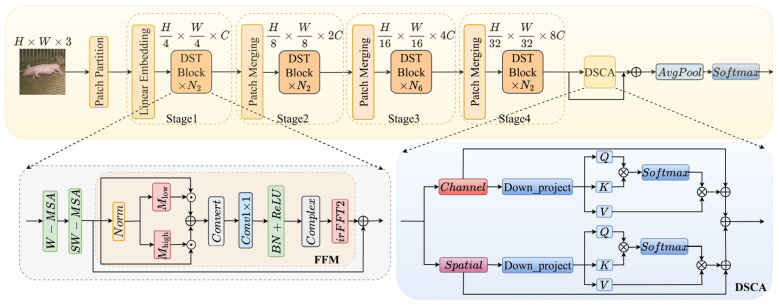
The overall architecture of DST. Based on the hierarchical Swin Transformer, the framework incorporates two parallel paths: a frequency-domain path and a spatial-domain path with enhanced channel modeling.

We propose a Dual-path Swin Transformer (DST) framework for accurate pig behavior recognition. This framework consists of two complementary paths: a frequency-domain path and a spatial-domain path with enhanced channel modeling. More precisely, by embedding the Frequency-domain Fusion Filter Module (FFM) and the Decoupled Spatial-Channel Attention (DSCA) mechanism into the end-to-end architecture, the framework enhances the behavior-discriminative feature representation and strengthens the modeling of cross-dimensional dependencies. An overview of the framework is shown in Figure 1.

The overall process is as follows. Let the input feature tensor be *x*∈ℝ^*B*×*L*×*C*×*H*×*W*^, where *B* is the batch size, *L* is the number of frames, *C* is the number of channels, and *H* and *W* are the height and width of the feature map, respectively. The spatial feature tensor at each stage of the Swin Transformer and the frequency-domain feature output by the FFM are processed in parallel to enhance the representation of low- and high-frequency image features. The features output from the four stages of Swin Transformer are fused along the channel dimension to obtain the fused feature. Subsequently, the fused feature is input into the *DSCA* mechanism to further optimize feature dependencies. Then, mean pooling (AvgPool_*L*_) is applied along the temporal dimension *L*, and the resulting feature is normalized by Softmax to obtain the pig behavior classification probability *Y*. Finally, the model outputs the corresponding behavior label.

### 2.1 Frequency-domain fusion filter module

**Figure 2:**
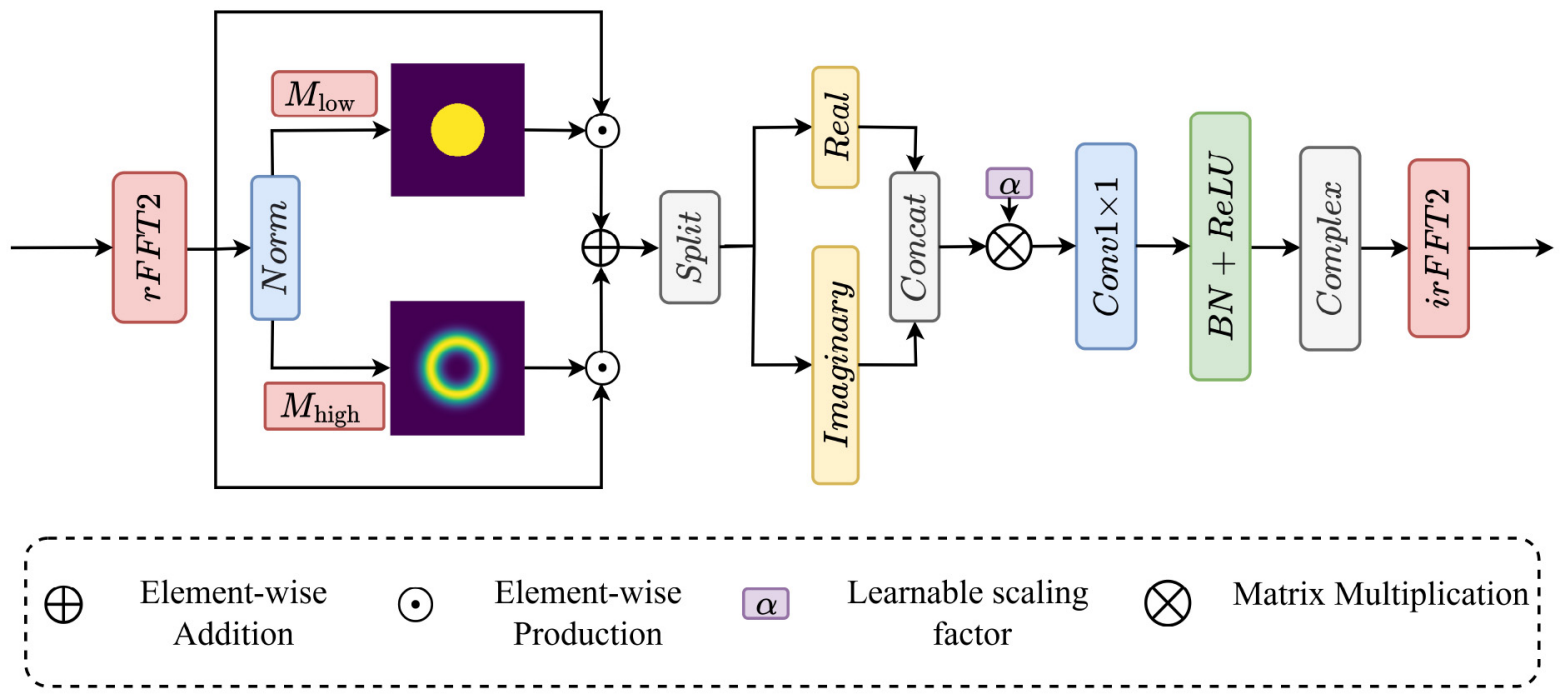
The FFM architecture diagram.

To enhance the anti-interference capability of the model in complex pigpen environments, this study proposes the Frequency-domain Fusion Filter Module (FFM). The module uses the Ideal Low-pass Filter to preserve low-frequency global structural information of the coarse-scale pig body and effectively suppress high-frequency background interference. The Gaussian High-pass Filter enhances detail features through smooth frequency transitions and reduces the influence of low-frequency illumination variations on feature representation. The parallel connection of the two forms a complementary frequency selection mechanism to achieve joint enhancement of global structures and local details. The architecture of the FFM is shown in Figure 2.

The specific implementation of FFM is as follows: First, to transform spatial-domain features into the frequency domain while preserving energy conservation, an orthonormal two-dimensional real-valued Fast Fourier transform is adopted:


$$\begin{array}{l} F\left(x\right)=\text{rFFT}2\left(x\right) \end{array}$$ (1)

where *F*(*x*) denotes the frequency-domain features after transformation, and the rFFT2 preserves the $L_{2}$ norm to stabilize the training procedure. To clarify the physical meaning of each frequency point, the frequency coordinates are normalized. The normalized frequency coordinates are given by:


$$\begin{array}{l} u\left(h\right)=\frac{h}{H}-\frac{1}{2},\;h=0,1,\ldots ,H-1 \end{array}$$ (2)

$$\begin{array}{l} v\left(w\right)=\frac{w}{W},\;w=0,1,\ldots ,W^{\prime }-1 \end{array}$$ (3)

where $W^{\prime }=\left\lfloor \frac{W}{2}\right\rfloor +1$ denotes the width of the non-redundant spectrum produced by rFFT2. Because rFFT2 retains only the non-negative frequencies along the width dimension, *v* is defined on the one-sided interval [0, 0.5], whereas *u*∈[−0.5, 0.5).

Based on this normalized non-redundant spectrum, the radial coordinate for mask construction is defined as


$$\begin{array}{l} R\left(u,v\right)=\sqrt{u^{2}+v^{2}} \end{array}$$ (4)

where *R*(*u, v*) denotes the radial coordinate of each frequency location in the normalized non-redundant spectrum.

The core of frequency-domain filtering is to selectively emphasize high- and low-frequency components by modulating the frequency spectrum with masks. Ideal low-pass filtering (*R* ≤ ρ) retains low-frequency global structural features via hard-threshold screening, which is implemented as:


$$\begin{array}{ll} M_{low}\left(u,v\right) & =\{\begin{array}{cc} 1, & R\left(u,v\right)\leq \rho \\ 0, & \text{otherwise} \end{array} \end{array}$$ (5)

In contrast, Gaussian high-pass filtering suppresses low-frequency responses near the DC component and progressively preserves high-frequency local detail features through a smooth radial transition:


$$\begin{array}{l} M_{high}\left(u,v\right)=1-\exp\left(-\frac{R{\left(u,v\right)}^{2}}{2\sigma^{2}}\right) \end{array}$$ (6)

where σ>0 controls the radial transition width of the Gaussian high-pass response.

The filtered frequency-domain representation is obtained by summing the masked low-frequency and high-frequency responses:


$$\begin{array}{l} X=F_{filtered}\left(x\right)=M_{low}\left(u,v\right)⊙F\left(x\right)+M_{high}\left(u,v\right)⊙F\left(x\right) \end{array}$$ (7)

where ⊙ represents element-wise multiplication.

### 2.2 Decoupled spatial-channel attention mechanism

**Figure 3:**
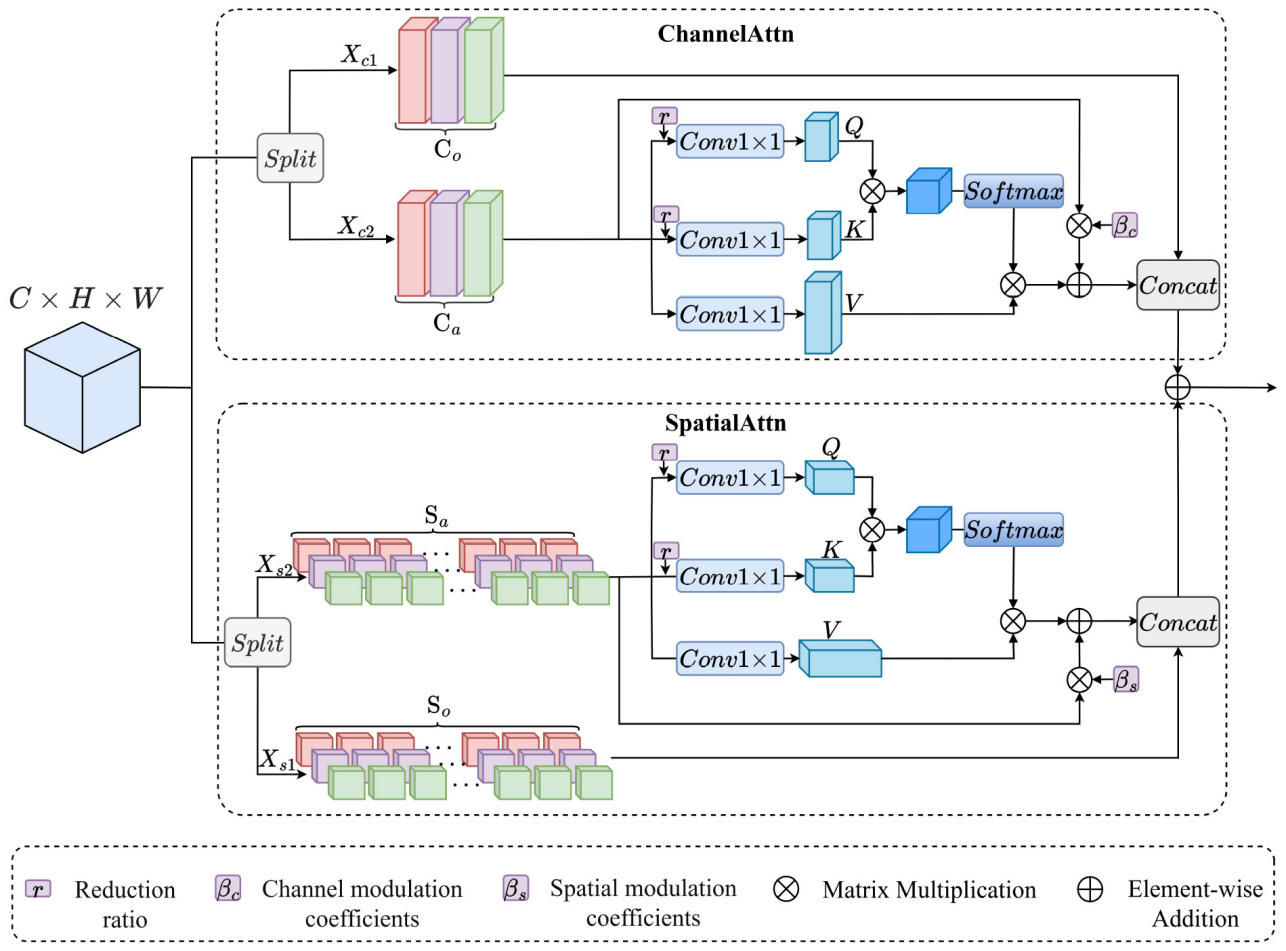
The DSCA mechanism architecture diagram.

To accurately model spatial and channel dependencies and reduce redundant information interaction, we propose a Decoupled Spatial-Channel Attention (DSCA) mechanism. It decomposes the attention modeling process into two independent branches for channel feature enhancement and spatial feature enhancement respectively, and the overall output is the sum of the two branches. In detail, the spatial branch adaptively emphasizes key body parts associated with pig behaviors, while the channel branch strengthens behavior-related feature channels. This decoupled design enables more precise modeling of cross-dimensional feature dependencies and enhances the discriminative representation of pig behaviors, as shown in Figure 3.

#### 2.2.1 Channel attention branch

The ChannelAttn Branch models interdependencies among channels. By dynamically focusing on critical channel features, it enhances informative feature representation while avoiding redundant computation.

Given the input feature tensor *X*∈ℝ^*B*×*L*×*C*×*H*×*W*^ and a learnable scaling parameter λ (initialized to 0.5), this parameter enables the model to dynamically allocate computational resources according to the complexity of the input and adaptively adjust the proportion of channels processed by the attention mechanism.


$$\begin{array}{l} \begin{array}{cccc} C_{\text{a}} & =\max\left(1,\left\lfloor \lambda \times C\right\rfloor \right),\; & C_{\text{o}} & =C-C_{\text{a}} \end{array} \end{array}$$ (8)

where ⌊·⌋ denotes the floor function, ensuring that *C*_*a*_ is an integer. The feature tensor is partitioned along the channel dimension into an original feature retention subset *X*_*c*1_ and an attention modulation subset *X*_*c*2_:


$$\begin{array}{l} X_{\text{c1}}=X\left[:,:,:C_{\text{o}},:,:\right],\;X_{\text{c2}}=X\left[:,:,C_{\text{o}}:C_{\text{a}}+C_{\text{o}},:,:\right] \end{array}$$ (9)

For the attention modulation subset, Query (*Q*), Key (*K*), and Value (*V*) feature branches are constructed via 1 × 1 convolutions. The dimensions of the query and key branches are reduced to *C*_*a*_/*r*, where *r* is the dimensionality reduction coefficient used to decrease computational complexity. Meanwhile, the number of channels in the value branch is maintained as *C*_*a*_ to preserve feature representation capability.


$$\begin{array}{lll} Q_{c} & = & {Conv}_{1\times 1}\left(X_{c2};C_{a}/r\right),\\ K_{c} & = & {Conv}_{1\times 1}\left(X_{c2};C_{a}/r\right),\\ V_{c} & = & {Conv}_{1\times 1}\left(X_{c2};C_{a}\right) \end{array}$$ (10)

For each sample within a batch, a correlation matrix is calculated over channels to quantify the strength of channel-wise dependencies. Subsequently, the attention weights are normalized via Softmax:


$$\begin{array}{l} X_{\text{c}}^{\prime }=Softmax\left(\frac{Q_{c}\cdot K_{c}^{T}}{\sqrt{C_{a}/r}}\right)\cdot V_{\text{c}}+X_{\text{c2}}\times \beta_{c} \end{array}$$ (11)

where β_*c*_ is a learnable modulation coefficient. Finally, the original *X*_c1_ and modulated subsets $X_{\text{c}}^{\prime }$ are concatenated along the channel dimension to preserve the input shape.

#### 2.2.2 Spatial attention branch

The SpatialAttn branch is complementary to the ChannelAttn branch in terms of the attended dimension. The core design models correlations between spatial positions. By locating key spatial regions, it enhances the representation of behavior-related local features.

Given the same input *X*, based on the learnable proportion parameter γ∈(0, 1), the number of spatial positions subjected to attention *S*_*a*_ and the number retaining original features *S*_*o*_ are determined, with the constraint that the attention scope contains at least one spatial position:


$$\begin{array}{l} S_{a}=\max\left(1,\left\lfloor \gamma \times H\times W\right\rfloor \right),\;S_{o}=H\times W-S_{a} \end{array}$$ (12)

The 5D feature tensor *X* is vectorized along the spatial dimension (*H*×*W*) to obtain a 4D tensor $X_{vec}\in {\mathbb{R}}^{B\times L\times C\times \left(H\times W\right)}$. Subsequently, the resulting tensor is partitioned along the spatial-position dimension into an original feature preservation subset *X*_*s*1_ and an attention-modulated subset *X*_*s*2_.


$$\begin{array}{l} X_{s1}=X_{vec}\left[:,:,:,:S_{o}\right],\;X_{s2}=X_{vec}\left[:,:,:,S_{o}:S_{o}+S_{a}\right] \end{array}$$ (13)

where *X*_*s*1_ preserves the first *S*_*o*_ spatial positions without attention modulation, while *X*_*s*2_ contains the remaining *S*_*a*_ positions for spatial dependency learning. For *X*_*s*2_, three feature branches (i.e., query, key, and value) are generated via 1 × 1 convolutions.

Next, Query (*Q*_*s*_), Key (*K*_*s*_), and Value (*V*_*s*_) are generated from *X*_*s*2_. The spatial attention weights are then normalized by Softmax, and the output feature $X_{s}^{\prime }$ is obtained under the modulation of the learnable coefficient β_*s*_. Finally, $X_{s}^{\prime }$ is concatenated with the initial *X*_*s*1_ to produce the output feature.

## 3 Dataset and implementation details

### 3.1 Dataset

**Figure 4:**
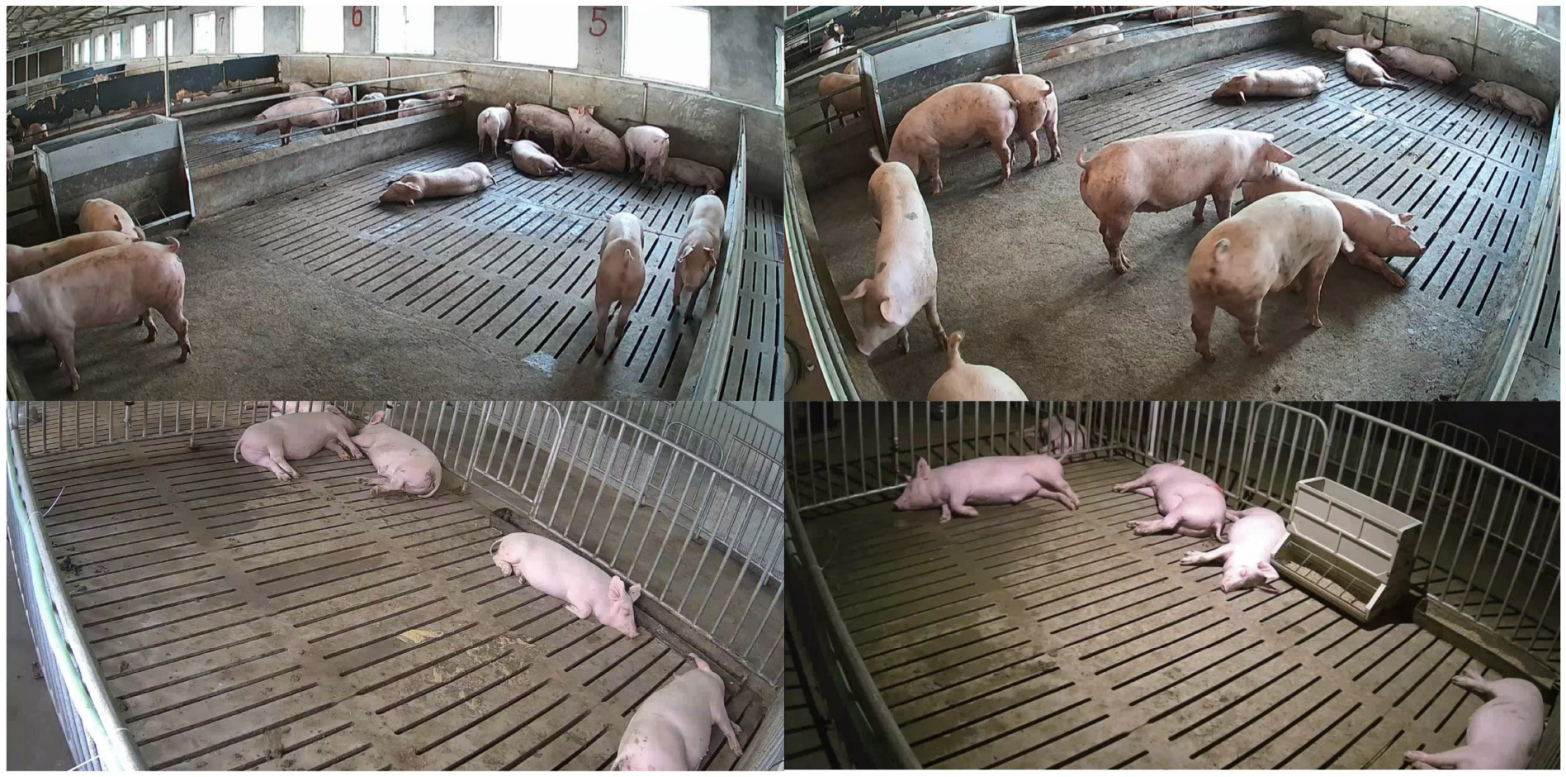
Representative surveillance camera images captured inside the pigpen.

The dataset was collected from the pig breeding base of Nonglvyuan Agricultural Co., Ltd. in Xiangfen County, Linfen City, Shanxi Province, from August 12 to September 25, 2022. Data collection was conducted in six pigpens, each housing about ten six-month-old three-way crossbred pigs. All pens were equipped with Hikvision DS-2DE3Q120MY-T/GLSE cameras mounted on the side wall, 3 meters above the ground, facing the pigpen at a 45-degree depression angle. The cameras captured RGB color space videos with a resolution of 1,920 × 1,080 pixels and a frame rate of 10 frames per second. Representative surveillance camera images captured by the installed cameras inside pigpen are presented in Figure 4.

During the data collection phase, approximately 1.5 TB of raw video data was obtained, containing over 5,000 video files. Effective behavioral segments of 5 to 10 seconds were extracted to build a standardized dataset containing 2,755 videos. The dataset covers the six pig behavior categories mentioned above, with a similar number of samples in each category and an overall balanced distribution. Each category contains approximately 450 video segments. Each video consists of 10 chronologically ordered frames forming a complete segment. To improve data quality, the inter-frame difference method was used to detect blurred frames, and 3.7% of low-quality segments in the original data were removed. The videos were collected under real pigpen conditions, where environmental disturbances such as pig vocalizations, fan noise, fan vibration, and motor operation may exist. Since the proposed DST framework uses only RGB surveillance video frames as input, acoustic noises do not directly enter the model or directly affect the frequency-domain filtering process. The proposed Frequency-domain Fusion Filter Module operates on visual feature maps rather than on acoustic or vibration signals. However, mechanical vibration may indirectly affect visual quality by causing camera jitter, motion blur, or image instability. Therefore, blurred and unstable segments were removed during data cleaning to reduce the influence of low-quality visual inputs. Finally, all samples were divided into a training set and a validation set at an 8:2 ratio. The data for each category are shown in Table 1.

**Table 1:** Distribution of video samples across behavior categories in the training and validation sets.

Behavior category	Walking	Eating	Drinking	Lying	Exploring	Fighting
Training sample	354	391	340	379	388	350
Validation sample	89	98	86	95	97	88

The dataset constructed in this study includes six types of pig behaviors: Fighting, Drinking, Exploring, Walking, Eating, and Lying. These behaviors were selected because they are closely related to pig welfare, activity status, feeding and drinking conditions, social interaction, and potential health abnormalities. They cover both social behaviors and essential survival-related behaviors.

**Figure 5:**
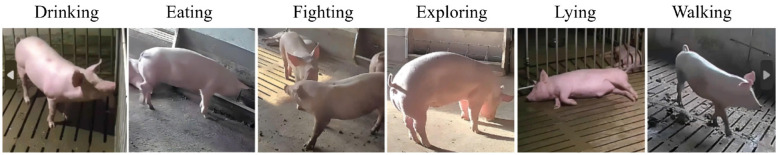
Example frames illustrating the six pig behavior categories from the dataset.

The visual judgment criteria for each behavior category were defined according to observable posture, body movement, and the spatial relationship between pigs and pen facilities. Specifically, Fighting involves physical aggressive interactions, such as pushing, biting, chasing, or body collision. Drinking is characterized by oral contact with the nipple drinker and sucking. Exploring includes sniffing or nosing the pen floor, walls, or objects. Walking refers to locomotion accompanied by alternating limb movements and continuous body displacement. Eating involves head insertion into the feeder and chewing. Lying encompasses resting postures in which the body remains in contact with the floor, with limbs either stretched or curled. Examples of each category are shown in Figure 5.

These behavior categories also have practical significance for pig health and welfare monitoring. Walking and Exploring reflect the activity level and environmental adaptation of pigs; Fighting is associated with aggression, stress, and potential injury risk; Eating and Drinking are directly related to feed and water intake status; and Lying reflects the resting state of pigs and may provide cues for abnormal health conditions when the duration or frequency of this behavior deviates from normal patterns. Therefore, accurate recognition of these behaviors is meaningful for welfare assessment, production management, and early abnormality detection in pig farming.

### 3.2 Implementation details

This section describes the experimental settings for model training and evaluation, including the hardware and software environment and the main training parameters. To improve training stability and generalization, the Exponential Moving Average (EMA) strategy is adopted during training. Hardware and software configurations are listed in Table 2.

**Table 2:** Hardware and software configurations for the experiments.

Term	Configuration
Operating system	Ubuntu 20.04.6
GPU	NVIDIA TITAN Xp (12 GB GDDR5X)
CPU	Intel i7-7800X (3.50 GHz)
CUDA version	11.8
Deep learning framework	PyTorch 2.2.0
Python version	3.10.18
NumPy version	1.26.4
RAM	125 GB
Optimizer	AdamW
Learning rate	2e-5
Batch size	4
Epochs	150
Input resolution	224 × 224
Input shape	[4, 10, 3, 224, 224]
EMA decay	0.9999

### 3.3 Loss function

For training the proposed DST model, the standard cross-entropy loss is adopted. The loss is defined as follows:


$$\begin{array}{l} Loss=-\frac{1}{B}\overset{B}{\underset{i=1}{\sum }}\overset{K}{\underset{k=1}{\sum }}y_{i,k}\log p_{i,k} \end{array}$$ (14)

where *B* and *K* denote the batch size and the number of categories, respectively. *y*_*i, k*_ denotes the ground-truth label of the *k*-th category for the *i*-th sample, and *p*_*i, k*_ denotes the predicted probability of the corresponding category.

### 3.4 Performance evaluation

To evaluate the classification performance of the proposed model, four metrics are adopted in this study: Accuracy (ACC), Precision (P), Recall (R), and F1-score (F1). Accuracy represents the ratio of correctly predicted samples to the total number of samples. Precision, Recall, and F1-score are computed separately for each category, and the overall Precision, Recall, and F1-score are reported as the macro-averaged values across all categories.

For a given category, Precision and Recall are defined as:


$$\begin{array}{l} P=\frac{TP}{TP+FP} \end{array}$$ (15)

$$\begin{array}{l} R=\frac{TP}{TP+FN} \end{array}$$ (16)

where *TP*, *FP*, and *FN* denote the numbers of true positive, false positive, and false negative samples, respectively, for the considered category.

The F1-score for the considered category is defined as:


$$\begin{array}{l} F1=2\times \frac{P\times R}{P+R} \end{array}$$ (17)

The overall Precision, Recall, and F1-score are obtained by averaging the corresponding category-wise results across all categories.

## 4 Experiments and analysis

This section presents a quantitative evaluation of the proposed DST framework. Comparisons with state-of-the-art methods are provided, followed by an ablation study to clarify the contribution of each enhancement path.

### 4.1 Performance comparison of different models

To evaluate the superiority of the DST model in pig behavior recognition, we compared it with several mainstream vision models, including ViT (23), Swin Transformer (24), ConvNeXt (15), EfficientViT (28), and TransNeXt (29). The comparison results are shown in Table 3.

**Table 3:** Overall performance comparison of different models on the validation set.

Model	Accuracy (%)	Loss	Precision (%)	Recall (%)	F1-score (%)	Params	FLOPs
ViT	92.41	0.2796	92.61	92.35	92.42	49.8M	11.2G
Swin Transformer	93.49	0.2801	93.50	93.46	93.45	27.5M	4.5G
ConvNeXt	92.95	0.2801	93.01	92.97	92.95	28.5M	4.5G
EfficientViT	93.31	0.2968	93.34	93.32	93.27	24.0M	2.1G
TransNeXt	93.67	0.2588	93.69	93.67	93.67	28.2M	5.4G
DST	**94.94**	**0.2499**	**94.95**	**94.92**	**94.91**	33.0M	5.3G

Bold values indicate the best-performing results in the corresponding comparison; for loss, lower values indicate better performance.

The results in Table 3 show that DST achieves the best overall performance among the compared models. Notably, DST achieves an accuracy of 94.94%, which outperforms those of ViT, Swin Transformer, ConvNeXt, EfficientViT, and TransNeXt by 2.53, 1.45, 1.99, 1.63, and 1.27 percentage points, respectively. It also achieves the lowest loss value among the compared models, at 0.2499. Furthermore, DST attains a precision of 94.95%, a recall of 94.92%, and an F1-score of 94.91%, corresponding to maximum improvements of 2.34, 2.57, and 2.49 percentage points over the other models, respectively. These findings provide strong evidence for the superior performance of DST in pig behavior recognition. In terms of model complexity, DST maintains moderate parameter and computational costs, achieving the best performance with 33.0 M parameters and 5.3 GFLOPs, thereby striking a favorable balance between computational efficiency and model accuracy.

**Figure 6:**
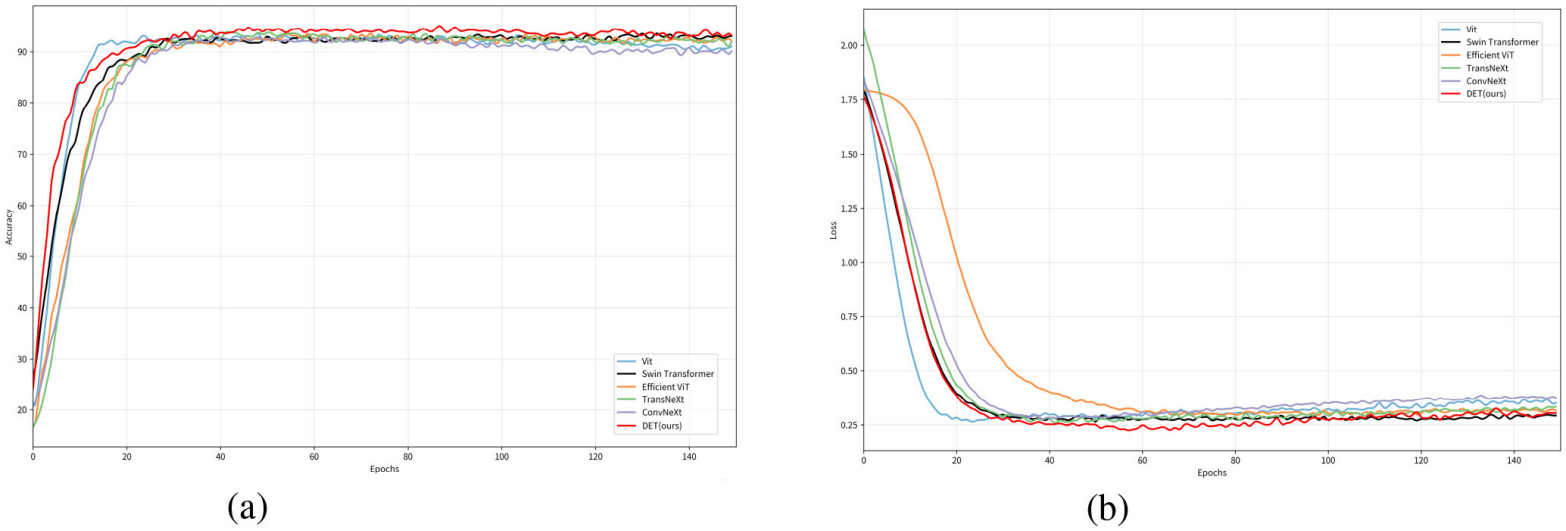
Validation accuracy **(a)** and validation loss **(b)** curves for different models. **(a)** Accuracy curve. **(b)** Loss curve.

Figure 6 shows the validation accuracy and validation loss curves of all models during training. As presented in Figure 6, DST outperforms other models in both accuracy and loss. More precisely, Figure 6a indicates that the accuracy of DST is consistently higher than that of the compared models after 30 training epochs. A performance lead is maintained by the DST model throughout the middle and late stages of the experiment, and the highest accuracy is ultimately achieved. In Figure 6b, the loss curve of DST shows a steady downward trend and converges to the lowest value among all compared models. These results further verify the effectiveness and stability of DST in complex pig behavior recognition tasks.

To further assess the category-wise recognition performance of different models, we compare their results across six pig behavior categories, namely Drinking, Eating, Fighting, Exploring, Lying, and Walking. For each category, Precision, Recall, and F1-score are reported, together with the corresponding macro-averaged results, namely Avg Prec, Avg Rec, and Avg F1. The detailed results are shown in Table 4.

**Table 4:** Comparison of recognition performance across six pig behavior categories for different models.

Model	Metric	Category						Avg Prec	Avg Rec	Avg F1
		Drinking	Eating	Fighting	Exploring	Lying	Walking			
ViT	Precision	97.5	95.88	**95.24**	87.63	95.92	83.51	92.61	92.35	92.42
	Recall	90.70	94.9	90.91	87.63	98.95	**91.01**			
	F1-score	93.98	95.38	93.02	87.63	97.41	87.1			
Swin Transformer	Precision	97.62	**96.94**	92.39	87.88	96.88	89.29	93.50	93.46	93.45
	Recall	95.35	96.94	96.59	89.69	97.89	84.27			
	F1-score	96.47	**96.94**	94.44	88.78	97.38	86.71			
ConvNeXt	Precision	98.8	96.88	90.11	92.31	93.94	86.02	93.01	92.97	92.95
	Recall	95.35	94.9	93.18	86.6	97.89	89.89			
	F1-score	97.04	95.88	91.62	89.36	95.88	87.91			
EfficientViT	Precision	97.62	95.96	91.3	91.21	92.16	91.76	93.34	93.32	93.27
	Recall	95.35	96.94	95.45	85.57	98.95	87.64			
	F1-score	96.47	96.45	93.33	88.3	95.43	89.66			
TransNeXt	Precision	**98.81**	95.92	92.22	90.43	**97.92**	86.81	93.68	93.68	93.67
	Recall	**96.51**	95.92	94.32	87.63	98.95	88.76			
	F1-score	**97.65**	95.92	93.26	89.01	**98.43**	87.78			
DST	Precision	98.80	95.96	94.51	**92.63**	95.96	**91.86**	**94.95**	**94.92**	**94.91**
	Recall	95.35	**96.94**	**97.73**	**90.72**	**100**	88.76			
	F1-score	97.04	96.45	**96.09**	**91.67**	97.94	**90.29**			

Bold values indicate the best-performing results in the corresponding comparison; for loss, lower values indicate better performance.

Table 4 details the precision, recall, and F1-score of each behavior category. Overall, the DST model achieves the optimal average performance across all categories. Its average precision, recall, and F1-score reach 94.95%, 94.92%, and 94.91%, respectively, which are 1.27, 1.24, and 1.24 percentage points higher than those of TransNeXt, the second-ranked model. For specific behavior categories, DST performs prominently in Fighting behavior recognition, with 97.73% recall and 96.09% F1-score, both exceeding those of the other compared models. It also presents distinct advantages in recognizing Exploring and Walking behaviors with subtle motion differences, achieving corresponding precision of 92.63% and 91.86% and F1-scores of 91.67% and 90.29%. A 100% recall rate is obtained in Lying behavior recognition. The performance metrics of DST for Drinking and Eating behaviors are comparable to those of other models. These results further demonstrate the effectiveness of DST in multi-category pig behavior recognition.

**Figure 7:**
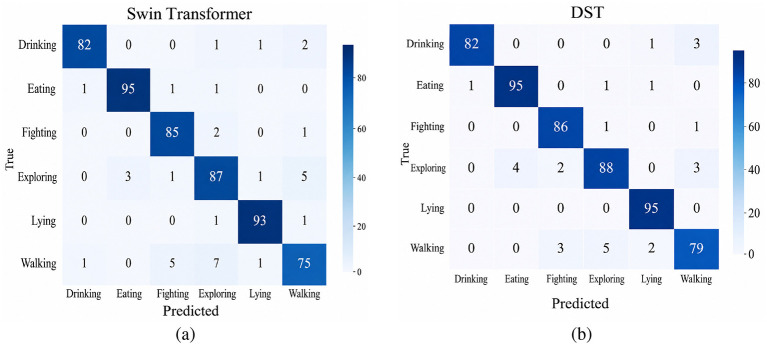
Confusion matrix comparison between the baseline Swin Transformer and the proposed DST model on the validation set. **(a)** Confusion matrix of the baseline Swin Transformer. **(b)** Confusion matrix of the proposed DST.

The confusion matrices in Figure 7 further show the recognition performance of the baseline Swin Transformer and the proposed DST model across six pig behavior categories. Compared with the baseline, DST presents a more concentrated diagonal distribution, indicating reduced inter-class confusion. In particular, the diagonal entries for Lying, Walking, Fighting, and Exploring are improved, suggesting that DST achieves more accurate recognition for several behavior categories. The confusion between Walking and Exploring is also reduced, which is important because these two behaviors involve similar posture transitions, limb movements, and continuous spatial displacement in complex pigpen environments. These results provide visual evidence that DST improves fine-grained behavior discrimination under complex pigpen conditions.

The excellent performance of the DST model in pig behavior recognition can be attributed to two key modules. Coarse-scale global structural features such as the overall pig contour are extracted by the FFM using Ideal Low-pass Filter. Meanwhile, fine-grained high-frequency details including limb edges are preserved via Gaussian High-pass Filter. This enables the model to accurately capture subtle differences in limb movements between the Walking and Exploring behaviors. The spatial attention branch of the DSCA mechanism is able to precisely locate key limb regions of pigs, and its channel attention branch strengthens feature channels related to actions. The collaboration of the two branches enhances the model's ability to distinguish subtle behaviors in complex scenes.

### 4.2 Ablation experiments and module effectiveness analysis

To verify the effectiveness of the FFM and the DSCA mechanism in the DST model, ablation experiments are conducted based on the Swin Transformer baseline. The proposed modules are gradually integrated into the baseline to evaluate their individual roles.

#### 4.2.1 Effectiveness of the frequency-domain fusion filter module

To investigate the effectiveness of the complementary design of different frequency-domain filtering strategies and the Frequency-domain Fusion Filter Module (FFM), we conduct ablation experiments. Four single filtering strategies and their different combinations are compared, and the results are shown in Table 5.

**Table 5:** Ablation study of the FFM.

Index	Ideal low-pass	Gaussian low-pass	Ideal high-pass	Gaussian high-pass	Accuracy (%)	Loss
1	×	×	×	×	93.49	0.2801
2	✓	×	×	×	94.03	0.2755
3	×	✓	×	×	94.03	0.2923
4	×	×	✓	×	94.39	0.2617
5	×	×	×	✓	94.03	0.2793
6	✓	×	✓	×	94.21	0.2859
7	✓	×	×	✓	**94.58**	0.2741
8	×	✓	✓	×	94.21	0.2662
9	×	✓	×	✓	93.85	0.2891

Bold values indicate the best-performing results in the corresponding comparison; for loss, lower values indicate better performance.

Table 5 summarizes the performance of various frequency-domain filtering strategies. Compared with the baseline Swin Transformer (index 1), which achieves an accuracy of 93.49%, the introduction of single-filter configurations (indices 2 to 5) increases the accuracy to a range of 94.03% to 94.39%. Among the single-filter configurations, the Ideal High-pass Filter achieves the best performance. In the joint filtering configurations (indices 6 to 9), the highest accuracy of 94.58% is achieved by the parallel operation of the Ideal Low-pass Filter and Gaussian High-pass Filter (index 7). This yields a maximum improvement of 0.73 percentage points over other paired-filter combinations. The loss of this configuration decreases to 0.2741.

**Figure 8:**
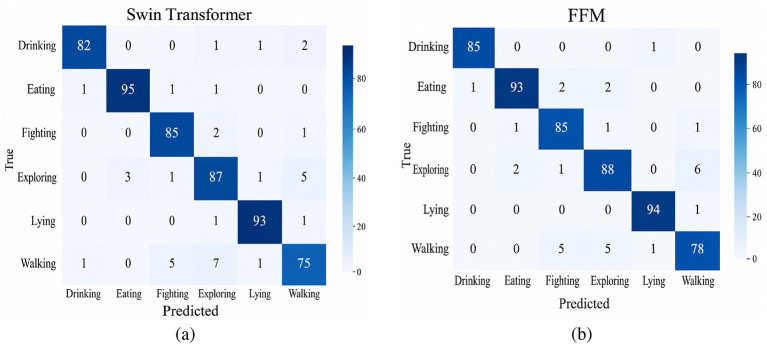
Confusion matrix comparison between the baseline Swin Transformer and the model equipped with the proposed FFM on the validation set. **(a)** Confusion matrix of the baseline Swin Transformer. **(b)** Confusion matrix of Swin Transformer with FFM.

The confusion matrices in Figure 8 further illustrate the recognition performance of the baseline Swin Transformer and the model equipped with FFM across six pig behavior categories. Compared with the baseline Swin Transformer, the model equipped with FFM shows a more concentrated diagonal distribution, indicating reduced inter-class confusion. In particular, the diagonal entries for Drinking, Lying, Exploring, and Walking are improved, suggesting that FFM improves recognition for several behavior categories. The confusion between Walking and Exploring is also alleviated, with fewer Walking samples misclassified as Exploring. This indicates that FFM helps preserve fine-grained local detail features and global structural information of the pig body, which are important for distinguishing behaviors with similar locomotion patterns and spatial displacement. Overall, these results provide visual evidence that the frequency-domain enhancement introduced by FFM improves recognition performance across behavior categories under complex pigpen conditions.

In the single-filter configurations, the Ideal High-pass Filter achieves the optimal performance. It effectively retains high-frequency details such as pig limb edges through hard-threshold truncation. As a result, the model's ability to distinguish fine-grained behavioral differences is enhanced. In contrast, the Ideal Low-pass Filter mainly preserves low-frequency global structures, such as the coarse overall contour of the pig body. It can hardly highlight the high-frequency local features related to behavior recognition. The Gaussian High-pass Filter keeps high-frequency information with smooth frequency transitions. However, when used alone, its enhancement effect on edge details is less pronounced than that of the Ideal High-pass Filter. Therefore, the Ideal High-pass Filter shows clearer advantages in the single-filter configuration.

In the joint filtering configurations, the combination of the Ideal Low-pass Filter and the Gaussian High-pass Filter outperforms single-filter configurations because the two filters provide complementary frequency-domain information. The Ideal Low-pass Filter preserves the global structural information of the pig body through hard thresholding, thereby improving robustness to background interference and partial occlusion. The Gaussian High-pass Filter enhances the representation of limb edges and posture-related local details through smooth frequency transitions. It also reduces the interference caused by low-frequency illumination variations, thereby improving the model's ability to discriminate fine-grained behavioral features. Their complementary effects improve behavior recognition performance in complex pigpen environments with uneven illumination, occlusion, and background interference.

#### 4.2.2 Effectiveness of the decoupled spatial-channel attention mechanism

To investigate the individual roles of the ChannelAttn and SpatialAttn branches in the DSCA mechanism, as well as their complementarity, ablation experiments were conducted in this section. Concretely, the channel attention branch and the spatial attention branch were introduced into the model already equipped with FFM for comparative evaluation. The experimental results are shown in Table 6.

**Table 6:** Ablation experiments on the DSCA mechanism.

ChannelAttn	SpatialAttn	Accuracy (%)	Loss
×	×	94.58	0.2741
✓	×	94.76	0.2216
×	✓	94.58	0.2616
✓	✓	94.94	0.2499

As shown in Table 6, introducing only the ChannelAttn branch increased the accuracy from 94.58% to 94.76%, while reducing the loss from 0.2741 to 0.2216. When only the SpatialAttn branch is introduced, the accuracy remained at 94.58%, whereas the loss decreased to 0.2616. When the two branches are simultaneously employed in parallel, the accuracy further improved to 94.94%, and the loss decreased to 0.2499.

This superior performance of the DSCA mechanism is mainly attributed to two factors. ChannelAttn enhances feature representations related to pig behavior discrimination by modeling inter-channel correlations and adaptively adjusting channel responses. SpatialAttn adaptively focuses on key pose regions such as trunk contours by modeling spatial position dependencies. When the two mechanisms are applied together, complementary enhancement is achieved in both the channel and spatial dimensions. As a result, behavior-related features are strengthened, and key body regions and postural cues are highlighted. This enables the model to capture cross-dimensional feature correlations and enhance its discriminative ability for pig behavior recognition.

#### 4.2.3 Effectiveness of FFM and the DSCA mechanism

To comprehensively assess the contributions of FFM and DSCA mechanism to the overall model performance, Swin Transformer is adopted as the baseline model. Fusion ablation experiments are then conducted by separately introducing FFM, DSCA mechanism, and their combination. The results are presented in Table 7.

**Table 7:** Ablation experiment integrating FFM and the DSCA mechanism.

FFM	DSCA	Accuracy (%)	Loss
×	×	93.49	0.2801
✓	×	94.58	0.2741
×	✓	94.03	0.2554
✓	✓	94.94	0.2499

As shown in Table 7, introducing only FFM increases the accuracy from 93.49% to 94.58%, while reducing the loss from 0.2801 to 0.2741. With the introduction of only the DSCA mechanism, the accuracy rises to 94.03%, whereas the loss drops to 0.2554. When the two modules are jointly employed, the accuracy further improves to 94.94%, and the loss falls to 0.2499.

Table 7 demonstrates that FFM and the DSCA mechanism are highly complementary. FFM enhances feature representation in the frequency domain while retaining both the global features of pig bodies and the edge details of their limbs. By using decoupled spatial and channel branches, the DSCA mechanism highlights key behavioral regions of pigs and discriminative feature channels. The collaborative use of these two modules effectively improves the overall performance of pig behavior recognition in complex scenes.

## 5 Discussion

This section aims to elucidate the underlying mechanisms responsible for the performance gains of DST in complex pigpen environments. To this end, the discussion is conducted from two perspectives: the adaptability and complementarity of frequency-domain filtering strategies, and the role of DSCA mechanism in modeling cross-dimensional dependencies.

### 5.1 Scenario adaptability and complementary effects of frequency-domain filtering strategies

In complex pigpen environments, multi-source interference can affect feature representation, thereby limiting the performance of pig behavior recognition. Therefore, the importance of frequency-domain filtering strategies lies not only in emphasizing information from specific frequency bands, but also in coordinating the preservation of global structural information and the enhancement of local detail features according to task requirements. On this basis, different filtering strategies are introduced to strengthen low-frequency structural information and high-frequency discriminative cues, thereby improving the model's robustness against interference in complex scenes.

Experimental results show that the parallel configuration of the Ideal Low-pass Filter and the Gaussian High-pass Filter achieves the highest accuracy. This result can be attributed to the complementary effects of the two filters in terms of their frequency response characteristics. Cluttered backgrounds and local occlusions easily disrupt the stable representation of the overall structure of the pig body. To address this issue, the Ideal Low-pass Filter suppresses high-frequency background interference through hard-threshold truncation while preserving the low-frequency global structural information of the pig body. As a result, the subject feature representation is stabilized, and the influence of local occlusion is mitigated.

On the other hand, the accurate discrimination of behaviors such as Walking, Exploring, and Fighting depends on fine-grained postural cues contained in the high-frequency components, such as limb edges and body contour boundaries. The Gaussian High-pass Filter enhances these detail features through smooth frequency transitions. It not only reduces the influence of low-frequency illumination variations on feature representation, but also avoids the ringing artifacts that may be introduced by hard-threshold truncation, thereby providing more stable local discriminative features for pig behavior recognition.

When only one type of filter is applied, the model usually strengthens only one aspect of feature representation. As a result, overall structural stability and local detail discriminability cannot be effectively balanced. In contrast, the parallel design of the Ideal Low-pass Filter and the Gaussian High-pass Filter fully exploits their complementary effects in frequency selection. Therefore, this combination outperforms both single-filter configurations and same-type filter combinations.

### 5.2 Decoupled spatial-channel attention mechanism for cross-dimensional dependency modeling

In complex pigpen environments, pig behavior recognition is affected not only by occlusion, illumination variations, and background interference, but also by insufficient collaborative modeling of key body regions and behavior-discriminative features. To address this issue, the DSCA mechanism is introduced to decouple cross-dimensional dependency modeling into a spatial branch and a channel branch, thereby enhancing key body regions and discriminative feature channels, respectively. In detail, the spatial branch emphasizes key body regions, such as trunk contours, limb positions, and other posture-related areas, thereby improving the model's perception of behavior-related local posture cues. The channel branch adaptively reweights feature channels highly correlated with pig behaviors, thereby strengthening the representation of subtle differences among behavior categories.

### 5.3 Potential applications of DST in smart pig farm management

From the perspective of practical deployment, the proposed DST framework can serve as a visual perception module for pigpen surveillance videos. By classifying video clips into six behavior categories, namely Walking, Eating, Drinking, Lying, Exploring, and Fighting, DST produces recognition results that can be summarized over time and converted into behavioral indicators, such as eating and drinking frequency, movement intensity, lying duration, exploratory activity, and fighting frequency. These indicators provide useful evidence for precision feeding management, welfare assessment, health monitoring, and future applications in reproductive management.

Eating and Drinking behaviors are closely related to feed and water management. Their frequency and duration can help evaluate feed and water intake patterns within a pen. Reductions in Eating or Drinking, persistent crowding around feeders or nipple drinkers, or imbalanced access to resources may indicate feed competition, insufficient resource allocation, or changes in intake conditions. Therefore, the recognition results generated by DST can support precision feeding management and focused farm inspection.

The recognized behaviors can also support welfare assessment and health monitoring. Changes in Lying, Walking, and Exploring may reflect variations in rest, activity level, and environmental adaptation, whereas Fighting may indicate increased aggression, stress, or potential injury risk. When combined with individual identification and tracking information, these behavior records may further support health warning information for individual pigs and timely inspection.

The current behavior categories also provide a basis for future extension to reproductive management. Walking and Exploring can serve as indirect behavioral cues for changes in activity rhythm during estrus periods. Fighting can reflect social or aggressive interactions in breeding groups. Eating, Drinking, and Lying can help characterize intake and rest patterns before or after artificial insemination, during farrowing management, or in other reproductive events. With future integration of reproductive records, individual pig records, and identity information obtained through individual tracking, DST outputs could more reliably support estrus monitoring, artificial insemination timing, farrowing management, and analysis of abnormal reproductive events.

## 6 Conclusion

This study proposes a Dual-path Swin Transformer (DST) framework for pig behavior recognition in complex pigpen environments. The framework embeds the Frequency-domain Fusion Filter Module (FFM) and the Decoupled Spatial-Channel Attention (DSCA) mechanism into a hierarchical Swin Transformer backbone. Specifically, the FFM enhances frequency-domain features by jointly extracting global structural information and local detail features. The DSCA mechanism decouples the attention modeling process into spatial and channel branches to adaptively enhance key regions and behavior-related feature channels, respectively. Experimental results demonstrate that DST achieves a recognition accuracy of 94.94%, outperforming the baseline Swin Transformer by 1.45 percentage points. These results validate the effectiveness of DST for pig behavior recognition in complex pigpen environments.

Future research will focus on adaptive feature enhancement, cross-scene generalization, and lightweight design. Concretely, adaptive frequency-domain filtering and spatial-channel attention modulation will be explored to further improve the model's robustness and discriminative capability in complex environments. In addition, multi-source data and domain adaptation methods will be incorporated to enhance cross-scene generalization. Furthermore, lightweight design and knowledge distillation strategies will be introduced to reduce model complexity while maintaining recognition performance.

## Data Availability

The complete raw video dataset is not publicly available due to farm privacy, data ownership restrictions, and its large size. Additional data may be obtained from the corresponding author upon reasonable request and with permission from the data owner.

## References

[1] Papakonstantinou GI Voulgarakis N Terzidou G Fotos L Giamouri E Papatsiros VG. Precision livestock farming technology: applications and challenges of animal welfare and climate change. Agriculture. (2024) 14:620. doi: 10.3390/agriculture14040620

[2] Rault JL Bateson M Boissy A Forkman B Grinde B Gygax L . A consensus on the definition of positive animal welfare. Biol Lett. (2025) 21:20240382. doi: 10.1098/rsbl.2024.0382 39837489 PMC11883819

[3] Bernabucci G Evangelista C Girotti P Viola P Spina R Ronchi B . Precision livestock farming: an overview on the application in extensive systems. Ital J Anim Sci. (2025) 24:859–84. doi: 10.1080/1828051X.2025.2480821

[4] Moreira MdR Trabachini A Amorim MdN Harada ÉdS da Silva MA Silva-Miranda KOd. The perception of Brazilian livestock regarding the use of precision livestock farming for animal welfare. Agriculture. (2024) 14:1315. doi: 10.3390/agriculture14081315

[5] Reza MN Ali MR Haque MA Jin H Kyoung H Choi YK . A review of sound-based pig monitoring for enhanced precision production. J Animal Sci Technol. (2025) 67:277. doi: 10.5187/jast.2024.e113 40264534 PMC12010234

[6] Marić K Gvozdanović K Djurkin Kušec I Kušec G Margeta V. Smart pig farms: integration and application of digital technologies in pig production. Agriculture. (2025) 15:937. doi: 10.3390/agriculture15090937

[7] Yang Q Chen M Xiao D Huang S Hui X. Long-term video activity monitoring and anomaly alerting of group-housed pigs. Comput Electr Agric. (2024) 224:109205. doi: 10.1016/j.compag.2024.109205

[8] Reza MN Lee KH Habineza E Kyoung H Choi YK Kim G . RGB-based machine vision for enhanced pig disease symptoms monitoring and health management: a review. J Animal Sci Technol. (2025) 67:17. doi: 10.5187/jast.2024.e111 39974778 PMC11833201

[9] Wu Y Zhou S Wu Z Chen Z Hu X Li J. Accelerated data engine: a faster dataset construction workflow for computer vision applications in commercial livestock farms. Comput Electr Agric. (2024) 226:109452. doi: 10.1016/j.compag.2024.109452

[10] Reza MN Kabir MS Haque MA Jin H Kyoung H Choi YK . Instance segmentation and automated pig posture recognition for smart health management. J Animal Sci Technol. (2025) 67:677. doi: 10.5187/jast.2024.e112 40519624 PMC12159697

[11] Reza MN Ali MR Kabir MSN Karim MR Ahmed S Kyoung H . Thermal imaging and computer vision technologies for the enhancement of pig husbandry: a review. J Animal Sci Technol. (2024) 66:31. doi: 10.5187/jast.2024.e4 38618025 PMC11007457

[12] Khotthada S Matthujak A Khamphakdi P Glinubon J Siriboon C Salangam I . Development and performance analysis of an electromagnetic needle-free jet injection device for efficient drug delivery in pig farms. Eng Sci. (2024) 33:1329. doi: 10.30919/es1329

[13] Wang G Guo Y Yu Y Shi Y Ying Y Men H. ColorNet: An AI-based framework for pork freshness detection using a colorimetric sensor array. Food Chem. (2025) 471:142794. doi: 10.1016/j.foodchem.2025.142794 39793357

[14] Lei K Tang X Li X Lu Q Long T Zhang X . Research and preliminary evaluation of key technologies for 3D reconstruction of pig bodies based on 3D point clouds. Agriculture. (2024) 14:793. doi: 10.3390/agriculture14060793

[15] Liu Z Mao H Wu CY Feichtenhofer C Darrell T Xie S. A convnet for the 2020s. In: Proceedings of the IEEE/CVF conference on computer vision and pattern recognition (2022). p. 11976–11986. doi: 10.1109/CVPR52688.2022.01167

[16] Pan Y Zhang Y Wang X Gao XX Hou Z. Low-cost livestock sorting information management system based on deep learning. Artif Intell Agric. (2023) 9:110–26. doi: 10.1016/j.aiia.2023.08.007

[17] An Y Yi Y Han X Wu L Su C Liu B . A hybrid attention-guided ConvNeXt-GRU network for action recognition. Eng Appl Artif Intell. (2024) 133:108243. doi: 10.1016/j.engappai.2024.108243

[18] Xu S Zheng H Tao S Chai Y He Q Chen H . A lightweight pig face recognition method based on efficient mobile network and horizontal vertical attention mechanism. IEEE Trans Instrum Meas. (2024) 73:1–14. doi: 10.1109/TIM.2024.3374313

[19] Kim D Heo B Han D. DenseNets reloaded: paradigm shift beyond ResNets and ViTs. In: European Conference on Computer Vision. Springer (2024). p. 395–415. doi: 10.1007/978-3-031-72646-0_23

[20] Wang Y Xi W. Uniconvnet: expanding effective receptive field while maintaining asymptotically Gaussian distribution for convnets of any scale. In: Proceedings of the IEEE/CVF international conference on computer vision (2025). p. 20922–20933. doi: 10.1109/ICCV51701.2025.01945

[21] Lou M Yu Y. Overlock: an overview-first-look-closely-next convnet with context-mixing dynamic kernels. In: Proceedings of the IEEE/CVF conference on computer vision and pattern recognition (2025). p. 128–138. doi: 10.1109/CVPR52734.2025.00021

[22] Wang Y Deng Y Zheng Y Chattopadhyay P Wang L. Vision transformers for image classification: a comparative survey. Technologies. (2025) 13:32. doi: 10.3390/technologies13010032

[23] Dosovitskiy A. An image is worth 16x16 words: transformers for image recognition at scale. arXiv preprint arXiv:201011929. (2020).

[24] Liu Z Lin Y Cao Y Hu H Wei Y Zhang Z . Swin transformer: hierarchical vision transformer using shifted windows. In: Proceedings of the IEEE/CVF international conference on computer vision (2021). p. 10012–10022. doi: 10.1109/ICCV48922.2021.00986

[25] Fan Q Huang H He R. Breaking the low-rank dilemma of linear attention. In: Proceedings of the computer vision and pattern recognition conference (2025). p. 25271–25280. doi: 10.1109/CVPR52734.2025.02353

[26] Vo XT Nguyen DL Priadana A Jo KH. Efficient vision transformers with partial attention. In: European conference on computer vision. Springer (2024). p. 298–317. doi: 10.1007/978-3-031-73010-8_18

[27] Zhang Y Fan Q Huang H. Vision transformer with sparse scan prior. In: Proceedings of the 33rd ACM international conference on multimedia (2025). p. 3664–3672. doi: 10.1145/3746027.3755145

[28] Cai H Li J Hu M Gan C Han S. Efficientvit: multi-scale linear attention for high-resolution dense prediction. arXiv preprint arXiv:2205.14756. (2022).

[29] Shi D. Transnext: robust foveal visual perception for vision transformers. In: Proceedings of the IEEE/CVF conference on computer vision and pattern recognition (2024). p. 17773–17783. doi: 10.1109/CVPR52733.2024.01683

